# Moral reasons to edit the human genome: picking up from the Nuffield report

**DOI:** 10.1136/medethics-2018-105084

**Published:** 2019-01-24

**Authors:** Christopher Gyngell, Hilary Bowman-Smart, Julian Savulescu

**Affiliations:** 1 Department of Paediatrics, University of Melbourne, Melbourne, Victoria, Australia; 2 Murdoch Children’s Research Institute, Melbourne, Victoria, Australia; 3 Faculty of Philosophy, Oxford Uehiro Centre for Practical Ethics, Oxford, UK

**Keywords:** genetic engineering, autonomy, informed consent, distributive justice, enhancement

## Abstract

In July 2018, the Nuffield Council of Bioethics released its long-awaited report on heritable genome editing (HGE). The Nuffield report was notable for finding that HGE could be morally permissible, even in cases of human enhancement. In this paper, we summarise the findings of the Nuffield Council report, critically examine the guiding principles they endorse and suggest ways in which the guiding principles could be strengthened. While we support the approach taken by the Nuffield Council, we argue that detailed consideration of the moral implications of genome editing yields much stronger conclusions than they draw. Rather than being merely ‘morally permissible’, many instances of genome editing will be moral imperatives.

## Introduction

Genome editing technologies have developed rapidly in the last few years, and point to a future where we can precisely edit the human germline. The most powerful gene editing technology is the CRISPR (Clustered Regularly Interspaced Short Palindromic Repeats)-Cas9 system. CRISPR-Cas9 is found naturally in bacteria, where it functions as a defence against viruses by cutting viral DNA into small, non-functional fragments. In 2012, a team at UC Berkeley showed that CRISPR-Cas9 could be modified in the lab, so that it could target virtually any DNA sequence.^1^ This allows researchers to cut effectively any part of the genome. Furthermore, once a DNA strand is broken, the cell’s own repair mechanisms could be recruited to delete, add or modify the sequence. In April 2015, it was announced that CRISPR had been used to make edits in human embryos for the first time.^2^ In August 2017, researchers in the USA used CRISPR to correct a mutation in human embryos that leads to a fatal heart condition—with virtually no off-target mutations.^3^ In November 2018, Dr He Jiankui announced that he had used the CRISPR-Cas9 system to edit the genomes of twins Lulu and Nana, in an attempt to make them resistant to HIV.^4^ Although this has not been independently confirmed, if true, it would be the first use of genome editing for human reproduction. This attempt has largely been met with condemnation, including by one of the authors of this paper,^5^ due to its experimental nature and lack of consideration for the welfare of the children. Such research has generated wide debate about the ethics of genome modification.

Genome editing of germ cells (embryos, sperm and egg cells) was initially very controversial and caused some to call for an outright ban on this application.^6^ Despite this, there has been a broad consensus among expert bodies that genome editing in research is morally permissible (see table 1 for summary). However, genome editing for reproduction, a practice called heritable gene editing (HGE), has been much more contentious.

**Table 1:** Recent organisational or expert body statements on the ethics of heritable gene editing (HGE)*

Year	Body	Title	Key points
2018	Nuffield Council on Bioethics	Genome editing and human reproduction	Applications of HGE may be morally permissible if they are consistent with the *welfare* of the future person and social justice. This may allow for applications beyond preventing disease. Further research must be done into efficacy and safety and a broader societal debate must be facilitated, as well as ensuring adequate governance.
2018	Génome Québec & Centre of Genomics and Policy	Human genome editing: ethical and policy considerations	The modification of human germ cells and embryos should be permitted in a basic or preclinical research context, any future clinical germline modifications should be for *serious diseases only* (and criteria need to be outlined) and an inclusive approach to policymaking should be encouraged.
2017	National Academy of Sciences, Engineering and Medicine	Human genome editing: science, ethics and governance	If technical challenges relating to safety and efficacy were overcome, clinical trials for HGE should only be permitted if there is an absence of reasonable alternatives, *be restricted to genes that clearly cause or predispose to a severe disability or disease*, with conversion only to genes that currently exist in the population. Regulatory oversight and public debate is essential. No trials for purposes other than the prevention of disease should be permitted *at this time*, but public debate on the matter should be facilitated.
2017	American Society of Human Genetics (ASHG)†	Human germline genome editing: ASHG position statement	At this time, it is not appropriate to conduct HGE that results in human pregnancy. As long as there is appropriate oversight, there is no reason to prohibit or not use public funds for in vitro germline gene editing for research into clinical applications. Future clinical applications should only proceed if there is: (i) *a medical rationale*, (ii) strong evidence base, (iii) an ethical justification and (iv) a transparent public process.
2017	Opinion Group of the Bioethics and Law Observatory, University of Barcelona	Declaration on bioethics and gene editing in humans	A moratorium is useless in an international and competitive scientific world. Regulatory frameworks are needed to govern any clinical application of germline genome editing, and it should *not be used for enhancement*.
2017	Council of Europe	Recommendation 2115: the use of new genetic technologies in human beings	Deliberate germline editing in human beings would cross a line viewed as *ethically inviolable*. A regulatory framework needs to be developed and a broad and informed public debate should be fostered.
2017	Federation of European Academies of Medicine (FEAM)	The application of genome editing in humans	There are major ethical, safety and efficacy issues that must be resolved before clinical applications of HGE can be considered. There is no broad societal consensus in Europe. Generally, FEAM *does not support genome editing for non-medical interventions* (including somatic genome editing), but anticipates a wide-ranging public discussion on this matter.
2017	European Academies Science Advisory Council	Genome editing: scientific opportunities, public interests and policy options in the European Union	It would be *irresponsible* to proceed with germline editing until the numerous safety and efficacy concerns have been resolved. There is a need for an ongoing forum.
2017	American College of Medical Genetics and Genomics	Genome editing in clinical genetics: points to consider	There are technical concerns (eg, the risk of off-target effects or epigenetic effects that may affect many future generations) as well as ethical concerns (which variants that are to be edited needs to be discussed at a societal level).
2015	National Academy of Sciences, Engineering and Medicine	International summit on human gene editing	It would be *irresponsible* to proceed with any clinical use of germline editing unless and until (i) the relevant safety and efficacy issues have been resolved, based on appropriate understanding and balancing of risks, potential benefits and alternatives, and (ii) there is broad societal consensus about the appropriateness of the proposed application.
2015	Academy of Medical Sciences (UK)‡	Genome editing in human cells—initial joint statement	Ethical and regulatory questions must be debated before clinical applications of editing germ cells can be considered.
2015	Hinxton Group§	Statement on genome editing technologies and human germline genetic modification	When all safety, efficacy and governance needs are met, there *may be morally acceptable uses for HGE for reproductive purposes*. Further debate as to what specific uses are morally acceptable is required.
2015	American Society for Gene and Cell Therapy (ASGCT) and Japan Society of Gene Therapy (JSGT)	ASGCT and JSGT joint position statement on human genomic editing	As available genome editing technologies are inadequately understood, and because the results of such manipulation could not be understood for decades or generations, these safety and efficacy concerns are sufficiently serious to support a *strong stance against HGE*.
2015	Alliance for Regenerative Medicine¶	Do not edit the human germline	The scientific community is urged to engage in dialogue and put in place a *voluntary moratorium* to discourage human germline modification. This is due to scientific and ethical risks such as that permitting even unambiguously therapeutic interventions could start down a path towards non-therapeutic genetic enhancement.
2015	IGI Forum on Bioethics, Napa, California**	A prudent path forward for genomic engineering and germline gene modification	Any attempts at clinical applications of germline gene editing are *strongly discouraged.* Expert forums of scientists and bioethicists must be held to further discuss these issues. Transparent research should be encouraged and supported. A globally representative group of a wide range of stakeholders (including the general public and government bodies) should be assembled to further discuss these issues and make policy recommendations.
2015	International Society for Stem Cell Research (ISSCR)††	The ISSCR statement on human germline genome modification	Any consideration of applying nuclear genome editing to the human germline in clinical practice raises significant ethical, societal and safety considerations. Therefore, the ISSCR calls for a *moratorium on germline genome editing in clinical practice* (excepting mitochondrial replacement therapy), although in vitro research is supported.
2015	National Institutes of Health (NIH)	Statement on NIH funding of research using gene-editing technologies in human embryos	Safety, ethical and regulatory issues means that altering the human germline in embryos is ‘*a line that should not be crossed*’. NIH will not fund any use of gene editing in embryos. (N.B.: as of 2018, NIH is funding research into *somatic* cell genome editing).

*Organisational recommendations are included here. A search was conducted using a branched strategy, beginning with the use of search terms in databases such as PubMed and Google Scholar using search terms such as ‘human heritable genome editing’, ‘germline gene editing’, ‘genome editing embryos’, ‘germline engineering’, ‘human germline editing’+‘ethics’, ‘policy’ and/or ‘recommendations’. Furthermore, citations and references were examined to generate further results. A large number of papers were identified discussing these issues, including a number of recommendations. Results were limited to those from 2008 onwards (ie, the past decade at time of writing); all papers by individuals or groups of individuals not representing an organisation, body or conference were discarded, as well as empirical research into opinions on germline editing by specific sectors of society (eg, general public, healthcare professionals). The final group of results was limited to organisations whose remit includes human genetics, genetics policy, bioethics, reproductive technologies and/or emerging technologies. Organisational *responses* to statements and journal editorials were not included. This list is not exhaustive.

†Statement endorsed by UK Association of Genetic Nurses and Counsellors, Canadian Association of Genetic Counsellors, International Genetic Epidemiology Society, US National Society of Genetic Counselors, American Society for Reproductive Medicine, Asia Pacific Society of Human Genetics, British Society for Genetic Medicine, Human Genetics Society of Australasia, Professional Society of Genetic Counselors in Asia, and Southern African Society for Human Genetics. The Alliance for Regenerative Medicine (an international multistakeholder group, including commercial stakeholders), released a statement supporting this position.

‡Joint statement with the Association of Medical Research Charities, the Medical Research Council and the Wellcome Trust.

§A group consisting of an international collection of academics from disciplines such as the life sciences, philosophy, medicine and economics, from universities including John Hopkins, Oxford and London School of Economics.

¶A group consisting of commercial companies, research institutions, non-profit organisations and patient advocacy groups. Corporations comprise 73% of stakeholders and thus this organisation should be primarily considered to represent the views of corporations such BD Biosciences, GE Healthcare, GSK, MilliporeSigma, Novartis and Pfizer, among many others. They later released a statement supporting the ASHG position statement (see footnote 1).

**A large number of expert stakeholders attended and made this position statement, including the inventors of CRISPR-Cas9.

††A non-profit organisation encouraging research into the use of stem cells.

In July 2018, the Nuffield Council on Bioethics released its report ‘Genome editing and human reproduction: social and ethical issues’.^7^ The report is significant for advocating an approach to the assessment of HGE based on ethical principles rather than applications. Any particular use of HGE could be morally permissible, provided it was consistent with promoting individual welfare and social solidarity.

In this paper, we critically analyse the approach taken by Nuffield report and its conclusions.

In the first section, The Nuffield Council’s report on genome editing and human reproduction, we provide a detailed summary of the Nuffield Council’s approach. While we applaud the Nuffield Council report as a significant step forward in the debate, we argue there is room to build on and strengthen their approach. In the second section, Social harms and collective action problems, we suggest ways their guiding principles could be improved by removing an implicit asymmetry between the two principles. In the third section, Categorical limits and moral imperatives in HGE, we argue that the conclusions stated in the report do not go far enough. Some uses of HGE are not merely morally permissible but are moral imperatives, even beyond the treatment of disease. Finally, in the section Governance and public attitudes, we examine the implications of recent attitudinal research by the Pew Centre which shows that the general public may support HGE for treatment but not enhancement.

## The Nuffield Council’s report on genome editing and human reproduction

In 2016, following its report which looked at the ethical issues associated with genome editing more broadly (e.g., including food), the Nuffield Council of Bioethics formed a working group to ’examine ethical questions relating to the attempted influence of inherited characteristics in humans, in the light of the likely impact of genome editing technologies’. After 2 years of work, the working party released its report titled ‘Genome editing and human reproduction’.

The first two sections of the report contextualise HGE within its immediate potential role as a reproductive technology used by individuals, its possible future applications and the social context in which those applications might evolve.

The report states that genome editing in the context of reproduction must be situated against a background of increased genetic knowledge informing reproductive options. Increased knowledge about genetic differences has created an ’epistemic shift' revealing previous dichotomies between states of health and disease, and thus therapeutic and non-therapeutic applications, to be inadequate (p. 26). Even if one views HGE as only permissible within the context of ’therapeutic' applications, its position as a reproductive technology means that it cannot be viewed as straightforwardly therapeutic in the same way as other medical technologies. HGE is not therapeutic in the sense that nobody who is in a current state of disease is being treated, but nor is it straightforwardly preventative (because the risk could be addressed by not having children) (pp. 22–23). Genomic intervention in reproduction is distinct from other human applications because it deals with possible persons rather than existing persons; it must be viewed as a means of fulfilling reproductive desires rather than a means of preventing disease (p. 47, footnote 143).

The report goes on to say that genetic information (that can be acquired by a number of technologies) places new responsibilities on whether to act or not act on this information when considering reproduction. Prospective parents who wish to use this knowledge to avoid or ensure certain genetic variants in their offspring are seeking specific outcomes. They desire a specific kind of child, that is, one that is genetically related to them and does/does not have a specific trait, condition or characteristic (p. 23). Existing assisted reproductive technologies, such as pre-implantation genetic diagnosis, provide one way for parents to pursue such goals, but rely on a sufficient abundance of embryos. If this is not possible, HGE may be more acceptable than other means to achieve parenthood such as donated gametes, due to the strong preference many people have for genetically related offspring (p. 25).

The report next situates the development of genome editing within its technical possibilities and a social and political context. Here, the report highlights that genome editing should not be viewed in isolation as an ’innovation', but instead encourages us to consider what a society in which it is widely available might look like. It outlines various strategies by which HGE might be deployed, including at the zygote stage on embryos created through in vitro fertilisation (IVF), and the possibility of creating modified gametes from induced pluripotent stem (iPS) cells instead of editing embryos directly (pp. 37–39).

There are a number of situations identified in the report where HGE may be the only option to create a genetically related child. These include the cases of Y-chromosome defects, or dominant conditions where one parent is homozygous (p. 45). More likely scenarios where HGE may be necessary are those where the chance of an unaffected embryo would be low to very low. For example, one or both parents may be heterozygous for a dominant condition, or cases where there are multiple undesired independently sorting variants (p. 45). Although not mentioned in the report, another circumstance where HGE might be the only option to create healthy genetically related children is when dominant *de novo* mutations occur in the germ cell line, such as within spermatogonial stem cells.

The report even raises the possibility of using HGE to avoid complex conditions that are common in populations and are difficult to avoid through selective approaches, because of the large numbers of genes involved (p. 46). Looking forward to a future in which HGE is widely available, the report envisages a wide range of possible applications. These include increased immunity and resistance to disease, tolerance for adverse environmental conditions (such as that of space), superabilities or other various factors such as the ability to make vitamins rather than having to consume them (p. 47).

The report notes that the social and political drivers for the development of this technology may initially be the use of embryos for ‘basic’ research. However, researchers cannot be morally insulated from the ethical implications of further uses that might develop from applied research (pp. 48–49). The report supports responsible research and innovation, encouraging reflection that may counteract technological momentum (p. 49). The use of genome editing in human reproduction has the potential to be socially transformative, and policy and regulation will play a key role in how this transformation may play out. The report identifies three kinds of concerns about the integration of new technologies into the landscape: first, that we may simply ‘sleepwalk’ into a new world, due to uncontrolled technological momentum; second, that the technology may be subject to function creep, where its use expands in possibly morally troubling ways; and third, that we may be headed down a slippery slope with no reliable ethical or legal means to distinguish morally unacceptable applications from morally acceptable ones (p. 55). Identifying these concerns can help to recognise key points in the process where better governance and ethical reflection may play an even more important role.

The report’s approach to the ethical issues surrounding HGE is largely framed around the concept of human rights and interests, with a view to the production of general principles. It examines the ethical issues through the lens of three different kinds of interests: (i) individuals directly affected by the technology (the parents and the future person), (ii) society, particularly those who may collaterally affected in less immediate and direct ways (such as people with genetic conditions) and (iii) the interests of humanity in general and future generations (p. 59). This examination concludes that ‘none of the considerations raised yields an ethical principle that would constitute a categorical reason to prohibit heritable genome editing interventions’ (p. xviii).

The report observes that, in terms of the interests of individuals, the use of HGE must be balanced between the prospective parents' interests and the welfare of the future person that may result. Prospective parents often desire a child that is genetically related to them, for a variety of reasons, most of which are ‘felt as well as reasoned’ (p. 59). Nonetheless, it does not necessarily follow that these desires constitute any sort of moral claim to a genetically related child. However, the report looks at two reasons why individuals should be supported in their reproductive projects. The first is from the view that procreation is a good, with a naturalist perspective seeing it as an essential part of human function and flourishing or the satisfaction of a natural human desire. More moderately, a view of procreation as a good can come from a recognition that social arrangements favour procreation. However, as the report concludes, it is distinctly difficult to position procreation as a good in itself (pp. 62–63). The second reason people should be supported in their reproductive projects stems from a respect for procreative interests. These interests lead to both a negative right and a positive right, although the extent to which the latter is applicable is likely to be dependent on social context (pp. 63–64).

The reproductive interests of the parents must, however, be constrained by another set of interests: that of the future person. This, of course, raises issues such as the non-identity problem, which the report addresses in the following fashion. There are a number of possible children that exist in the form of mental images that the parents may have; as more and more decisions are made during the reproduction process, the diversity of these possible children narrows to become closer to the nature of the actual future child. These decisions can be about various properties of the child, and such properties can include those relating to the child’s levels of welfare. The parents bear responsibility for the state of affairs that result from their decisions, which carry moral weight (pp. 66–67). The report examines the extent and limits of this responsibility. It discusses arguments that have been put forward that (i) no genome editing is permissible (such as those autonomy-based arguments from Habermas), (ii) some genome editing is permissible (generally drawing a distinction between ’therapeutic' and ’enhancement' applications) and (iii) some genome editing is morally required (similar to the principle of procreative beneficence).

The report addresses a number of issues with all of these approaches. The first set of arguments raise the issue of genetic determinism, and the question of how genome editing can be sufficiently demarcated from other parenting strategies in a way that is morally coherent (p. 68). The second falls prey to the difficulty of distinguishing therapeutic applications from enhancement, including but not limited to disability rights and feminist critiques of the normativity implicit in any such argument (pp. 69–72). About the third, the report raises concerns about the application of this principle in practice, such as the ability to sufficiently and reliably identify genomic variants associated with welfare, and the risk of the burden of expectation (p. 72). However, all these approaches inform the first principle that the report formulates: the use of genome editing technologies must secure and be consistent with the welfare of any future person that may be born as a result of those technologies (p. 75). This principle is necessary but not sufficient for any application of HGE to be morally permissible.

### Principle 1: the welfare of the future person

Gametes or embryos that have been subject to genome editing procedures (or that are derived from cells that have been subject to such procedures) should be used only where the procedure is carried out in a manner and for a purpose that is intended to secure the welfare of and is consistent with the welfare of a person who may be born as a consequence of treatment using those cells.

It is notable that in the formulation of this principle, the Nuffield Council has specifically referenced a general approach focused on the *welfare* of the future person rather than any particular distinction between different applications of HGE, such as ‘therapeutic’ versus ‘enhancement’. As the report itself notes, it is very difficult to draw a clear distinction between therapeutic and non-therapeutic applications and this approach sidesteps this difficulty. The report explicitly states that there is no *a priori* reason that applications beyond the prevention of disease would not also be consistent with the welfare of the future person (p. 76). This is a significant step in opening the door for a variety of traits and characteristics to be considered for HGE. For example, this raises the possibility of actively including certain welfare-improving characteristics, rather than limiting HGE to the removal of welfare-diminishing characteristics.

The report goes on to address the interests of society, noting that reproduction takes place in a social context. Reproductive behaviours already can change the composition of a society without deliberate coordination, but reproductive technologies allow for this with greater certainty (p. 78). Other people in society may be collaterally and indirectly affected by the use of HGE. The report highlights questions surrounding diversity, shifting norms, disability critiques, social virtues and equity and justice.

The report notes that it is possible that genome editing could lead to changes in the level of diversity within the population—whether more or less would depend strongly on prevailing social factors (pp. 79–80). If the use of HGE becomes standard, this could lead to a shift in norms and the expectation of its use, which could decrease freedom. This can be reflected in current concerns around prenatal screening—what people *typically* do becomes what people *should* do, and thus what people feel pressured to do (pp. 80–82). This follows on to disability critiques, such as the expressivist objection, which states that attempts to deselect or prevent disability expresses or presupposes a negative view of people with a disability. However, the Nuffield Council explicitly rejects this objection when it comes to genetic conditions that significantly affect both quality and length of life (p. 82). Another set of critiques is that HGE represents attempts to overcome fragility and weakness, which are in themselves a valuable part of the human condition and should not be removed. However, the difficulty with these approaches is that a person would have to value such fragility enough that they would be willing to impart it on their own children (pp. 82–85). Finally, the report considers the importance of equity and justice and concludes that HGE should be restricted so as not to result in unfair advantages for certain groups of people (pp. 85–86). This leads on to the second principle formulated in the report (p. 87).

### Principle 2: social justice and solidarity

The use of gametes or embryos that have been subject to genome editing procedures (or that are derived from cells that have been subject to such procedures) should be permitted only in circumstances in which it cannot reasonably be expected to produce or exacerbate social division or the unmitigated marginalisation or disadvantage of groups within society.

The third set of interests that the report addresses is that of humanity and future generations. Potential adverse effects of HGE may only manifest themselves after several generations, and here the notion that we have responsibilities or moral obligations to future generations is key (p. 89). One perspective is that HGE could offer a means to remedy harms that have already been set in motion for future generations, such as runaway climate change (pp. 89–90). However, invoking the ‘precautionary principle’ would suggest that the uncertainty and possible negative consequences of HGE mean that it should not be applied. However, as the report notes, this constitutes no reason not to continue research and the development of the technology as a means of hedging bets against future events (pp. 90–91).

The report also addresses the relationship between HGE and transhumanism. Transhumanism is a concept that is closely linked to the emergence of technologies such as HGE. HGE could lead to the ‘self-overcoming’ of the human species to a grander, more capable species, and the report questions whether this constitutes a moral reason not to apply it. This may stem from a notion of a fundamental human dignity. HGE might be troubling because it threatens the integrity of human genetic inheritance (interference with the line of transmission that links the human family together) and the integrity of human genomic identity (distinguishing the human family from non-human beings) (p. 91). These concerns could be mitigated by replacing variants only with wild-type or typical variants, but of course the question of what is a wild-type or mutant variant is often value-laden in itself. The human genome is enormously varied, and many new variants are possible and likely to emerge. The report responds to this by stating that concerns about moving the human genome away from wild-type variants are prudential, rather than a categorical moral reason not to do so (p. 92). However, questions remain surrounding justice and equity, and the possibility of schisms between the ‘gene-rich’ and the ‘gene-poor’ (pp. 92–95).

The report concludes by stating that there are no categorical limits on the use of genome editing technologies, as long as (p. 97):

- They are not biologically reckless;
- They are consistent with the welfare of future people;
- They are not socially divisive;
- They are not initiated without prior societal debate.

We applaud the Nuffield Council’s approach to HGE and think it is an important step forward in the debate. A strength of the approach is that it outlines quite specific moral principles rather than merely appealing to broad concepts—such as earlier reports.

For example, the National Academiy of Sciences (NAS) report on genome editing endorses the principle of:

Promoting well-being: the principle of promoting well-being supports providing benefit and preventing harm to those affected, often referred to in the bioethics literature as the principles of beneficence and nonmaleficence.

It is left unspecified whose well-being we should be promoting through genome editing, what the components of well-being/harm are and exactly how that principle should apply to genome editing. Nearly all people would freely endorse this principle (and many of the other principles relied on in the National Academy of sciences report), but might draw radically different conclusions regarding genome editing. In contrast, the Nuffield Council’s first principle is much more specific and the scope and force is clear.

Nonetheless, there is room to improve and build from the Nuffield Council’s approach. In the next section, we show how an asymmetry in the structure between the two guiding principles leads to counterintuitive implications for an important set of possible uses for HGE, and suggest ways their second principle could be improved.

## Social harms and collective action problems

The first guiding principle adopted by the Nuffield Council concerns its effect on individuals. The report draws on the term ‘welfare’, which is explained as ’a broader concept than well-being ("being well", ie, "healthy"). In this sense, psychosocial welfare, and not just good health, is an important consideration’ (p. 76). Genome editing is only morally permissible if it is ’carried out in a manner and for a purpose that is intended to secure the welfare of and is consistent with the welfare of a person who may be born' (p. 75).

A relatively broad range of ways in which *individuals* could be harmed or wronged is discussed in the report. This includes the effects of genome editing on one’s physical health, and people’s psychological well-being, including any state ‘that might give the future person reasonable grounds to reprove their parents’ (p. 96).

In contrast, principle 2—which looks at the social effects of genome editing—is quite specific. Genome editing would be impermissible if it were ‘to produce or exacerbate social division or the unmitigated marginalisation or disadvantage of groups’ (p. 87). But this principle seems to overlook the fact that there are many ways in which society could be made worse that do not involve the creation of social division or marginalisation. If everyone in society were to be made significantly worse off, but in a way that did not increase inequality (or even possibly decreased it), we would still most likely view this as an undesirable state of affairs.

One strand of arguments in the literature on genetic enhancement centre on so-called ‘collective action problems’.^8–10^ In a collective action problem, one option is optimific from the perspective of an individual but results in collective harms if everyone pursues it. Imagine if it becomes possible to use HGE select for or against the predisposition to particular personality traits, such as extroversion. Extroversion is highly heritable^11^ and associated with increased levels of subjective well-being.^12^ Furthermore, recent studies have shown that mothers value extroversion in their children above other traits such as intelligence and conscientiousness.^13^ It is therefore plausible that were parents able to access HGE to increase their chances of having an extroverted child, they would use it. This would be consistent with both of the Nuffield Council’s guiding principles. As extroversion is associated with higher levels of subjective well-being, such a change would be consistent with the welfare of the child. While decreasing the frequency of introverts in society *might* lead to increased division and marginalisation as they become ‘the odd one out’, this is not necessarily so (they might become more highly prized as they become rarer). At any rate, the wrongness of such selection should not depend entirely on the contingent response of individuals to the decreasing features of a trait such as introversion.

If HGE were to dramatically increase the rate of extroverts in society, there is a sense that this would make society impersonally worse. Introverts contribute important forms of cognitive diversity which can benefit group problem solving.^14 15^ Having introverts in society can thus benefit many areas of life including achievement in the science and the arts.^16^

The ability of parents to target other characteristics through HGE, including height and innate immunity, could also lead to collective action problems.^8^ Such edits would be consistent with welfare of the child, yet if many people made those changes to their children, it could make society worse off in ways that do not necessarily involve increasing social division or marginalisation. If the average height of the population increased, this could increase the amount of resources that we use, and thus damage the environment. If many people selected similar immune genes for their children, this could leave us more susceptible to novel pathogens in the future.

This is not a criticism of the Nuffield Council’s report, as the type of HGE applications which could lead to collective problems are a long way off—and their consideration is not a pressing concern for the regulation of HGE now. However, reflection on them suggests ways in which the Nuffield report’s guiding principles could be improved. Namely, the second principle should evoke a broader concept of social harm, analogous to the concept of welfare in the first principle.

### Modified principle 2: social harms

The use of gametes or embryos that have been subject to genome editing procedures (or that are derived from cells that have been subject to such procedures) should be permitted only in circumstances in which it cannot reasonably be expected to produce or exacerbate social harms, including increased social division or the unmitigated marginalisation or disadvantage of groups within society.

While this principle loses some of the advantageous of specificity, it has the resources to respond to concerns relating to collective action problems.

## Categorical limits and moral imperatives in HGE

As stated above, the central conclusion of the Nuffield report on HGE was that there are no categorical limits on its use, provided applications are consistent with its guiding principles and preceded with broad public debate. We believe much stronger conclusions regarding the ethics of HGE can be drawn.

Technologies like HGE cannot be good or bad absolutely. We can speak of whether a particular *application* of a technology is good or bad, or whether their availability has good or bad effects on society—but technologies themselves are not the type of object to which the property of ‘good’ or ‘bad’ attaches.

The most basic ethical questions regarding HGE is therefore whether particular applications of it are good, bad, permissible, desirable, etc. In this section, we will examine some possible applications of HGE and show that rather than being merely morally permissible, some applications will be moral imperatives.

### Single gene disorders

A mark of success of medical genetics has been the diagnosis of the disease phenylketonuria (PKU) at birth. This is an inherited metabolic disorder in which levels of the enzyme phenylalanine hydroxylase are lowered. This means individuals cannot metabolise the amino acid phenylalanine. In 1962, a test was devised that allowed PKU to be diagnosed through a blood test.^17^ The ‘heel prick test’ is now routinely given to infants as part of newborn screening. Those children who are identified as suffering from PKU are put on a low phenylalanine diet or else they will develop severe intellectual disability. This diet means no bread, pasta, soybeans, egg whites, meat, legumes, nuts, watercress and fish. Such an environmental intervention is demanding. There is always a risk that foods containing phenylalanine will be consumed by mistake. The ubiquitous sweetener, aspartame, can cause a crisis.

Imagine an artificial enzyme was developed to replace phenylalanine. If this was administered regularly it would allow sufferers of PKU to consume a normal diet. Such a cure would be hailed as a breakthrough. There would be a moral imperative to provide this cure, just as there is an imperative to provide blood transfusion for severe bleeding, and antibiotics for infection.

Now imagine that instead of getting a pharmaceutical company to manufacture the enzyme, we could get the body to manufacture it. By altering the DNA of someone with PKU, we could get a patient’s own cells to produce the missing enzyme, phenylalanine hydroxylase. There are many advantages to not relying on pharmaceutical companies. Production inside the body allows for a more targeted response and more accurate dosages. Furthermore, it removes all chance that a patient would be unable to access the treatment, such as when the company has supply chain problems.^i^

Just as there would be a moral imperative to provide a replacement enzyme therapy for PKU, there would be an imperative to make safe genome edits which prevent PKU. If it becomes possible for carriers of the PKU mutation to prevent PKU in their future children through HGE, they will have an obligation to use this technology, in the same way they would have an obligation to use an enzyme replacement therapy.

#### Preimplantation genetic testing and HGE

The Nuffield report notes that in all but ‘extremely rare’ (p.44) cases, monogenic diseases like PKU can already be prevented through IVF in combination with preimplantation genetic testing (PGT), with the proviso that ‘it might not be reasonable to expect sufficient viable embryos with the characteristics sought to be available’ (p. 46). Let us try to put some numbers around the cases in which HGE would provide benefits over PGT in preventing single gene disorders due to a lack of viable embryos. In 2013 (the last year for which data are available), 18% of IVF cycles conducted in the UK produced only one viable embryo.^18^ So, for every 100 couples who go through IVF with the intention of using PGT to avoid disease, approximately 18 will produce a single viable embryo. In 2016 (the last year for which there is data), there were roughly 700 cycles of PGT for genetic disease in the UK.^19^ So, every year in the UK, around 126 IVF cycles are conducted for PGT and only produce one viable embryo. In these cases, it will not be possible to use genetic selection to avoid diseases. As people choose to attempt to conceive children later and later in life, in part for educational and career reasons, there will be a greater and greater scarcity of embryos.

The most common scenario in which couples use PGT is when they are both are carriers for recessive conditions. In these cases, there is a 25% chance that an embryo will carry both copies of the disease-predisposing mutation. This would imply that there are 31 cases in the UK per year in which HGE could avoid genetic disease in an embryo which PGT cannot. However, this is likely a conservative estimate. When parents are homozygous for dominant conditions like Huntington’s disease, or cases where there are multiple undesirable independently sorting variants, the number of affected embryos will be closer to 50%. One IVF company is on record as estimate that 48% of embryos which undergo PGT are affected by a genetic condition,^20^ although this will vary clinic to clinic.

Extrapolating from the above numbers would imply that, worldwide, there are several hundred cases a year where HGE would be the only option to produce unaffected offspring.

While several hundred cases a year can be considered rare, it is not negligible. If a public health measure could reduce the incidence of serious disease by several hundred a year, then we would have strong reasons to implement it. It would not merely be ‘morally permissible’ to take such a measure, but something that we actively ought to do. Of course, in situations of limited resources we have reasons to prefer interventions that maximise benefit, but this does not negate the moral reasons we have to benefit the few.

In sum, the application of HGE to prevent of single gene disorders is a good application of technology, and something we have moral reasons to pursue. If it were possible to use HGE to prevent single gene disorders, there would be a moral imperative to use it for this purpose. Of course, given this application alone may not benefit a large number of people, it may not justify using limited health resources developing HGE which could be spent on more effective health measures. But HGE also has potential to prevent far more common causes of disease, as we will explain in the next section.

### Polygenic diseases

Most diseases are not the result of just a few genetic changes. They are the result of many, sometimes hundreds, of genes combining together with environmental effects. Such polygenic diseases are among the world’s biggest killers. Cardiovascular disease is emerging as the biggest cause of death in the low-income and middle-income world. Together deaths from chronic diseases in those under 70 years are responsible for approximately 30% of all deaths worldwide.^21^ In addition to causing pain and death to individuals, chronic diseases place a huge burden on national health systems, consuming resources that could be used elsewhere. One study found that the healthcare cost associated with treating cardiovascular disease totalled €104 billion annually, for countries within the European Union.^22^

We know that there are genetic contributions to chronic diseases. Genome-wide association studies have identified at least 44 genes involved in diabetes^23^; 35 genes involved in coronary artery disease^24^ and over 300 genes involved in common cancers.^25^

It is possible to differentiate between individuals based on their genetic risk of developing chronic diseases. Using next-generation sequencing technologies (like whole genome or whole exome sequencing), polymorphisms occurring across many genes can be tallied and weighted giving an individual a ‘polygenic risk score’ that reflects their genetic predispositions to develop particular diseases and traits. Individuals can then be stratified into different risk categories (such as high risk, medium risk and low risk) based on their polygenic risk score.^26^

As genome editing technologies can target many genes at one time, it may become possible to use them to alter an individual’s polygenic risk score at the embryonic stage,^ii^ and shift individuals from a high-risk category to a low-risk category.iii,^iv^

Alternatively, it will be possible for individuals who know they have a high polygenic risk to particular diseases to use HGE to alter their gametes to ensure they do not pass this high risk on to their children.

For example, by editing around 27 mutations associated with coronary heart disease, it would be possible to reduce an individual’s lifetime risk by 42%^27^; by editing 12 genetic variants one’s lifetime risk of bladder cancer could be reduced by almost 75%.^28^

This application cannot be achieved through current methods of genetic selection. Say a couple want to use PGT to select for 15 different genes in an embryo, to reduce their likelihood of cardiovascular disease. Then they would need to create thousands of embryos to make it sufficiently likely that one will have the right combination at all 15 loci. The chance of the couple having such an embryo would be <1% with traditional IVF and PGD.^29^

Given the massive disease burden caused by chronic diseases, we have strong moral reasons to develop technologies that reduce their incidence—whether these operate through genetic or environmental mechanisms. Imagine scientists develop a new technology which potentially could be incorporated into exhaust filters, and would drastically reduce the amount of air pollution cars emit. In cities where cars are fitted with the exhaust filter, the incidence of respiratory disease would be decreased by 40%.

There are clearly strong moral reasons to develop this technology and pursue its applications. Developing the exhaust filter is not merely something it would be permissible to do, but something that there is an imperative to do. The very same reasons apply to the development of HGE.

One might respond that the clear difference between this case and HGE, is that HGE makes heritable changes and will thus affect future generations. However, air pollution is a known epigenetic modifier,^30^ that is, it makes changes to gene expression which can be inherited by future generations.^31^ Hence, reducing air pollution could also affect future generations. Of course, we need to consider what the long-term effects of any changes will be. But if the likely effect of a genetic change in one generation is to reduce risk of disease in future generations, this seems only to strengthen the case in favour of those changes.

If HGE could make genetic changes which reduce risks of polygenic disease in current and future generations, there would be an imperative to use it. Obviously, this application is a long way away from being plausible, possibly decades. One major difficulty is that we do not understand polygenic scores well enough to accurately predict the effects of large-scale changes. Still, we have moral reasons to develop HGE with the intention of using them for this purpose. First, it will reduce rates of premature death and disability due to chronic disease. Second, the use of HGE to make the highest risk individuals the same as the lowest risk individuals will be equality-promoting. Third, using HGE to lower the incidence of chronic disease will also promote justice. As stated above, health systems spend billions in resources to treat and prevent chronic disease. Using HGE in germline cells will probably be a relatively cheap way (in the proximity of US$20 000) of reducing someone’s susceptibility to chronic diseases. In a world of limited resources, taking a more expensive therapy has the opportunity cost of preventing the treatment of someone else’s disease. Justice requires we choose the most cost-effective option, other things being equal. If we do not invest in the most cost-effective option, we harm others who could use these resources.

### Enhancement

Just as polygenic scores could in theory be used to reduce rates of complex disease, they can target complex traits like intelligence.

General intelligence—the ability to learn, reason and solve problems—is the best known predictor of education and occupational outcomes.^32^

For decades, it has been known that around 50% of the observed variation in intelligence is due to genetic factors. A number of recent large studies have identified many polymorphisms, which help explain 20% of the heritable variation in intelligence.^32^

As with complex disease, using the polygenic scores it is possible to stratify the population into three board groups ‘high predisposition to high intelligence'; ‘medium predisposition to high intelligence’ and ‘low predisposition to high intelligence’. It will become theoretically possible to use HGE to shift individuals from the low or medium predisposition groups, into the high predisposition group.

Enhancing based on intelligence using polygenic scores would, in the words of the Nuffield report, be a form of enhancement that uses only ’wild-type’ variants (variants that already exist in the species) rather than a form of enhancing that goes beyond what currently exists in the species. In other words, it is a form of ‘normal range human enhancement’.^33^ While it may be possible in the future to enhance intelligence beyond levels that are currently observed in the species—such forms of enhancement are much less feasible at present.

Imagine a prenatal nutritional programme was developed, which was predicted to increase intelligence in children born with low innate predisposition to high intelligence. This would be seen as a breakthrough. We may soon be able to achieve the same with HGE.

One of the most intuitive concerns about technologies like germline engineering is the effect on equality. It is feared that germline engineering would only be available to the rich, and that it could widen the gap between rich and poor, adding biological advantages to already existing social ones. This is an important and complex issue, faced not just by genome editing but other goods like education. Ethically, we must take steps to ensure that the benefits and costs of HGE are evenly or fairly shared. As recognised by the Nuffield Council, this is not a reason to ban the technology, or fail to develop it, but a reason to ensure it is developed responsibly.

However, it is also possible to use HGE to directly improve equality, as the intelligence example shows. Nature is a biological lottery which has no mind to fairness. Some are born gifted and talented, others with short painful lives or severe disabilities. Currently, diet, education, special services and other social interventions are used to correct natural inequality. It may be that targeting combinations of genes is an effective means of promoting equality in education. For example, there are natural variations in people’s innate ability to learn how to read. This often matters little for people in higher socioeconomic groups, who can afford to spend extra time with their children teaching them how to read, or employ tutors, etc. However, for those in lower socioeconomic groups, this predisposition can leave them illiterate for life. While other measures could in theory no doubt remedy this inequity of outcomes, evening out the genetic starting point could prove the most effective way. This method would have the additional benefit of being passed to future generations. Genome editing could be used as a part of public healthcare for egalitarian reasons.

Boosting intelligence and other cognitive traits through HGE will be an ‘enhancement’, rather than disease prevention. As noted by the Nuffield report, this does not by itself reduce the moral reasons we have to pursue it. We have a moral imperative to use all reasonable means to produce equality in education.

### Future generations and intergenerational justice

One of the key interests considered by the Nuffield Council report is that of future generations. It is crucial that the very long-term consequences of developing or failing to develop HGE be considered. Humans often exhibit a cognitive bias towards the near future and neglect then how our actions may affect the very far future. This can distort our appraisal of technologies.

The obligations we have to future generations are often described in terms of intergenerational justice. We owe future generations the same considerations that we owe our contemporaries. We should not unnecessarily deplete the ozone layer, for example, if this will greatly harm future persons at an only small benefit to ourselves.

Some worry that by engaging in HGE we risk harming future generations by negatively altering our genome. There is no doubt that some application of HGE could harm future generations (e.g., see discussion of collective action problems in Section 3); however, such applications are not the inevitable consequences of the development of HGE, and can be mitigated or avoided.

Moreover, a deep engagement with the interests of future generations will show why there is strong moral imperative to develop HGE as a matter of intergenerational justice.^34^

Modern medicine is removing selection pressures that humans have historically been subjected to. This is increasing the rate of random mutations accumulating in the genome and poses a risk to future generations, as made clear by Michael Lynch in a 2016 article in the journal *Genetics*:

What is exceptional about humans is the recent detachment from the challenges of the natural environment and the ability to modify phenotypic traits in ways that mitigate the fitness effects of mutations, for example, precision and personalized medicine. This results in a relaxation of selection against mildly deleterious mutations, including those magnifying the mutation rate itself. The long-term consequence of such effects is an expected genetic deterioration in the baseline human condition, potentially measurable on the timescale of a few generations in westernized societies.^35^

As we develop effective and accessible treatments for disease, we all but guarantee that the incidence of those diseases will increase in future generations. This is because mutations which arise that contribute to those diseases are no longer selected against.

For example, short sightedness (myopia) has been historically very rare because it was selected against in hunter-gatherer societies.^36^ Modern technologies such as glasses, contact lenses and Lasik eye surgery help correct such vision problems. In modern societies, those with naturally poor eyesight have the same fitness as those who have naturally good eyesight. This allows deleterious mutations to occur in the genes which influence vision and not be selected against. Rates of myopia are now over 50% in many countries, making populations increasingly reliant on technology for this basic biological function. It is likely that reduced selection against poor vision has caused some of this increase. While it is easy to correct for myopia, the same process will allow mutations to accumulate in genes which influence other biological functions.

The percentage of people who require blood pressure medication,^37^ assisted reproductive technologies^38^ and have genetic predispositions to deafness, are all increasing. While social changes play a major role in these changes (eg, poor diet and sedentary lifestyle, delayed childbearing), biological factors also play an important part.^39 40^ In future generations, nearly all people may be reliant on technologies for these basic functions, as well as many others.

This will be bad for individuals, who become increasingly dependent on technologies for basic functions, and need to spend much of their time and money acquiring a range of therapeutic goods. Similarly, society will become burdened with spiralling healthcare costs. Furthermore, the consequence of natural disasters will become much more severe if people are reliant on a variety of complex technologies whose supply can be disrupted.

Fortunately, there is a way for our descendants to avoid such a medicalised future. Using HGE, we could edit out disease-causing mutations as they arise in our genome. This will allow our descendants to enjoy the same level of genetic health as we enjoy today.

Of course, many diseases have a lifestyle element—we have mentioned cardiovascular disease and infertility. Many resist using biological interventions to treat lifestyle problems. For example, it seems absurd to genetically modify human beings to be able to tolerate a diet consisting solely of foods with low nutritional value.

However, as we have argued, there are biological components to many contemporary diseases that are worthy of modification. Moreover, even if such diseases were entirely lifestyle or social in origin, which intervention we ought to choose—modifying the biological, psychological, social or natural environment—depends on the costs and benefits of the particular intervention, and relevant moral values. For example, it may be possible to prevent skin cancer by avoiding exposure to the sun, or by increasing the production of melanin, or by increasing the capacity of our immune cells to attack skin cancers. Which we should choose depends on the context.

A common assumption in environmental ethics is that we have obligations to members of future generations. According to one principle of intergenerational justice, ’existing generations ought not act so as to worsen the position of future generations by depleting non-renewable resources with no compensatory action or recompense’.^41^

It is clear that the use of modern medicine is worsening the position of members of future generation, by allowing random mutations to occur to our genome. Fortunately, there is a straightforward compensatory action—developing HGE. This is not something that is merely permissible—but a moral imperative.

## Governance and public attitudes

Ultimately, it is up to the public to make decisions about the ways genome editing can be applied. This is a guiding principle of liberal democracies. As noted by the Nuffield Council (p. 162), before any changes are made to the laws governing HGE, broad and inclusive public debate is necessary.

We endorse this view, but wish to add that public debates surrounding HGE need to be supplemented by public education initiatives. Making truly informed decisions about complex scientific matters requires people to understand science. A recent study by the Pew Study showed that 86% of Americans with high scientific knowledge approved of the use of HGE to prevent diseases that would be apparent at birth. This drops to 56% of people with low knowledge of science.^42^ Such research shows how familiarity with a subject matter shapes one’s view of it.

The Pew Research also shows a great divide between people who think HGE is permissible to prevent disease (72% for disease present at birth; 60% for later onset diseases) and those that think it is permissible for enhancement (18%). This is interesting because as noted by the Nuffield Council, there seems not to be essential reasons as to why the use of HGE to prevent disease is different than its use for human enhancement. What is important is that any use be consistent with promoting individual welfare, and does not negatively impact society.

Just as is the case with science, for people to make truly informed decision on *ethical* matters, ethical education is required. People should learn about concepts such as justice, freedom and well-being from an earlier age, and learn how to think critically about such topics. Only then can we truly make informed decisions about technologies like HGE.

## Conclusion

Genome editing technologies are developing rapidly, and so too is our understanding of their moral implications. The consensus of various expert bodies on the ethical implications of genome editing has shifted in response to greater engagement with the underlying philosophical issues. This has been exemplified by the recent Nuffield Council report, ‘genome editing and human reproduction’. Rather than drawing arbitrary lines between different possible uses of HGE, the Nuffield Council report engages with the fundamental ethical principles that should guide our appraisal of genome editing—concerns for individuals, for society as a whole and for future generations.

Nonetheless, we think deep engagement with underlying ethical issues of HGE yields much stronger conclusions than those drawn by the Nuffield Council. It will be ‘morally permissible’ to engage in HGE and will be morally ‘required’ in some instances.

The human genome was created by a blind process of mutation and selection occurring over thousands of generations. This process had no foresight for the creatures it would produce. This has resulted in vast natural inequality. The most extreme examples are single gene disorders, where some people become destined to a short life with much pain due to random quirks in their DNA. Others are born with high risks of chronic disease like heart disease and cancer. We ought to use powerful technologies like HGE to correct these inequalities and promote human flourishing. Such actions are moral imperatives.

## Footnotes

i It should be noted that the parents of the affected child will need to access HGE again if they want to ensure any future children they have are also unaffected. Given the cost associated with HGE, this may be seen as creating a division between parents who can and cannot afford it. However, such issues of access already exist for parents of children with PKU, which can cost around US$10 000 per year for the medical food and formula. HGE will certainly be much cheaper than the cost of treating PKU over a lifetime—it could thus help reduce such inequities. For a more complete discussions, see Gyngell *et al.* 43 We thank an anonymous reviewer for making us confront this issue.

ii This first require an embryo to be biopsied at very early stage (eg, two-cell or four-cell stage), to reduce risks of mosaicism.

iii This possibility was first brought to our attention by Roman Teo Oliynyk’s unpublished manuscript ‘Could future gene therapy prevent aging diseases?’^44^

iv In each of these case, there is a possibility that people in the low-risk group are overall worse off than those in the high-risk group, as the genes associated with high risk are beneficial in some other way. There will be a need for greater research into the overall effects of particular mutation before this application of HGE was undertaken.
